# Historical Sketches of Quarantine

**Published:** 1857-05

**Authors:** 


ART. II.—*Historical Sketches of Quarantine.* Address, delivered before the Philadelphia County Medical Society, January 28th, 1857; by WILSON JEWELL, M.D., President. Published by order of the Society. Pp. 30.

IN agreeable deviation from the usual custom of annual medical addresses, Dr. Jewell has chosen, for this discourse, a subject at least equal, in present interest, to those of medical ethics, or medical reform. It is indeed a topic "of magnitude, and world-wide importance." The view he has preferred to take of it is, as his title indicates, an historical one; dwelling, especially, upon the events of the early history of his own state and city.

The term quarantine (from *quaranta*, forty), is, we are told, although other opinions exist, "most likely to have had its origin in reference to the time of purification practised during the patriarchal ages." Both sacred and profane history give early instances of precautions used to prevent the spread of pestilential and contagious diseases.

"It was not, however, until the 14th century, that any attempts were made to enforce legal restrictions, as a means of protection against the introduction of disease. The return of one of the military expeditions undertaken by Christians for the recovery of the Holy Land, from the power of Infidels, brought with it, we are told, the Egyptian plague. Being looked upon as a new disease, it excited an unusual degree of attention. Its great mortality, and its contagious character, gradually attracted notice. It was soon discovered, that those who avoided the sick, escaped the disease. Building on this hypothesis, measures for defence were early instituted, to avoid all intercourse with persons affected with the disease, and to prevent all intercourse with places where the plague prevailed.

"The first of these regulations originated with Viscount Bernabo, of Reggio, in Italy, and was issued in January, 1374."* "In 1399, Viscount John imitated the example of his predecessor, when the disease appeared again, but in milder terms." "We have, however, no account of any well-defined legal code of regulations being introduced, until about the middle of the 15th century." The Venetian Senate, to exclude the plague, supposed to be brought from Constantinople and the Levant, "in 1448, instituted a quarantine by the enactment of a digest of laws, known, from that period to the present, as the laws of quarantine."

A few years subsequent to the passage of these laws, we have an account of the first organized Lazaretto, or pest-house. It was erected in 1453, on the Island of Sardinia, and called *Il Lazaretto Vecchio*. Another, erected in 1468, was entilled *Il Lazaretto Nuovo*. At a still later period, 1485, the Republic of Venice established the first board of health. It consisted of three nobles appointed by the Grand Council. They were called the Council of Health. In 1504, they received the right of life and death over those who violated their sanitary regulations; and no appeal was allowed from their decision.

From Venice, the quarantine system extended along the shores of the Adriatic, and, at last, to other commercial nations. It was enforced for the first time in England, about the year 1710, in Queen Anne's reign, when the plague was raging in towns on the Baltic Sea.

* The authorities of Florence are said to have used occasional precaution, as early as 1348.—*Russell on the Plague.*

"At that period, the greatest excitement prevailed. But in 1720, during the dreadful plague of Marseilles, France, the government taking the alarm, appointed the celebrated Dr. Richard Mead to execute a draft of quarantine regulations, with the hope that, if enforced, their country would be preserved from the pestilence then desolating one of the fairest cities of France. The Parliament approving the suggestions drawn up by this learned physician, repealed the act of 1710, which was defective, and passed an act establishing quarantines throughout the commercial ports of the kingdom, which law, until within a few years, has been enforced."

Twenty years before the enactment of this law of 1720, in England, a similar movement was projected in our own country, and in the province of Pennsylvania; under the auspices of its proprietary, William Penn. Philadelphia was visited, in 1699, having then a population of about 4000 souls, by a "pestilential fever, which carried off six, seven, and sometimes eight a day, for several weeks together; there being few, if any houses free from sickness." Although no medical account of it is extant, it is believed to have been the yellow fever, or Barbadoes distemper; and journalists state it to have been supposed to be "brought into the colony from the West Indies."

In the following year, 1700, a quarantine law was passed by the Assembly, being the first act of the kind on record in America. In Massachusetts, a health-law, partially quarantine, was enacted in 1701. In New York, the first act of the kind was passed in 1758.

It does not appear that this primitive health-law of Pennsylvania was enforced, with any strictness, for nineteen years at least after its enactment. The earliest instance on record in this State, if not in all America, of the detention of a vessel on account of sickness, that has any resemblance to the enforcement of quarantine laws, took place in Philadelphia, in 1728, in the case of the Ships Dorothy and Pharaoh.

"In the requirements urged in this case, there were many wholesome principles of sanitary police, not only understood, but carefully observed. Those sick on board the vessel were removed into fresh air and other conveniences. The bedding and all woollen goods were to be exposed on deck, and to be well aired at a convenient distance, before entering the city. The vessel, also, had to undergo a process of purification by smoking with tobacco and washing with vinegar."

"The next historical event on record, demanding the enforcement of quarantine, did not happen until 1738, ten years after the detention of the Dorothy. It was in the case of the two Ships, the Nancy and Friendship, from Rotterdam and Amsterdam."

The same year introduced proceedings which led, in 1743, to the establishment, near Philadelphia, of a Lazaretto, or, as then called, a Pest-house, or hospital, for the accommodation of sick emigrants upon their arrival in the province. This was, as Dr. Jewell remarks, the dawn of a noble charity; the origin of the first public hospital in Philadelphia. The above proceedings are detailed, with much interest, and many other facts of great local importance are given, in the address before us; but we must confine ourselves to a mere sketch of them.

In 1741, the yellow fever had for the second time visited Philadelphia. In January, 1744, a ship arrived from the Mediterranean, where plague was then prevalent. This arrival produced a great sensation; but no disaster followed.

In 1747, the yellow fever again returned, in an epidemic form; notwithstanding that "great care had been observed in the examination of all vessels from abroad." Several visitations of "Palatine Fever" are also enumerated, as in 1738, 1754, &c.; originating among emigrants, and probably typhous in its character; although the medical records leave this ambiguous.

No further action was taken by the Assembly in regard to quarantine laws until 1774, when a committee of the House was appointed to introduce a new bill "for the protection of the province from pestilential diseases in ships;" which was passed January 22d, 1774. This health law remained unaltered for nineteen years; during that time it was "almost, if not quite, a dead-letter law." The war of the revolution, by obstructing commerce, removed, or placed out of sight, the occasions for its observance. Since that time, no changes, important in principle, at least, have taken place in this State.

Leaving, for the present, the history of quarantine elsewhere (which, however, from some of his references, we conclude to have been discussed by the author in a work of greater extent, not yet published), Dr. Jewell concludes with some general remarks on its bearings upon public hygiène. To these we have looked with much interest. Few men have had ampler opportunities for the study of the subject; and very few have given it such industrious research. As an active member, and for a time, President of the Philadelphia Board of Health, Dr. Jewell has learned all that could be learned by actual and laborious observation, in a large manufacturing and commercial city, of the causes of disease, and of the modes of its prevention. Let us hear what he has concluded upon, in regard to the main subject of this discourse. Speaking of quarantine, he says:

"It involves the preservation of the health and life of individuals, as well as nations. It deserves the attention and study of statesmen, of philosophers, physicians, and of the humblest individual in society. If it has embarrassed commerce, and wasted millions of dollars, it has kept disease from our dwellings, and saved thousands of lives. If it has oppressed the few, it has conferred blessings upon the masses; and if it has failed to arrest the progress and spread of epidemics, it must be conceded that in numerous instances it has been useful by resisting the introduction of disease; hence it cannot be too highly appreciated, nor too thoroughly investigated."

After an allusion to the views of the London General Board of Health, and others, in opposition to quarantine, he continues :

"Unwilling as I am to commit my opinion to the extreme views embraced in this commission, or, on the other hand, to agree in sentiment with those ultraists, who, from a different stand-point, are disposed to regard the quarantine system as the only safe means to be relied upon for security from the invasion of pestilential and contagious diseases, nevertheless, I am deeply impressed with the fact, that quarantine, as it now exists, and wherever enforced, in this or in foreign countries, is not only inefficient and oppressive, but calculated to entail upon commerce unnecessary interruptions and unreasonable embarrassments.

"That in many instances they have failed to accomplish the laudable purpose for which they were originally instituted, history furnishes numerous melancholy examples. That the isolated means adopted to dissipate the morbific agent, and preserve the crew and passengers of ships in a healthy state, have proved the cause of a foul atmosphere, and the nourishment and development of the very evil it was designed to prevent, is alike true. The doctrine, too, of a specific contagion, or the spread of epidemic diseases by contagion, which was universally received when quarantines were first instituted, has, within the present century, undergone almost an entire revolution, especially in relation to those epidemics which for centuries, have occasionally made their appearance among the nations of the earth, depopulating their cities, and prostrating their commercial energies.

"A judicious modification of thepresent unsound, ill-advised, and ancient code of quarantine laws is, therefore, not only called for, but absolutely necessary. In the domain of the science of hygiene, quarantine has already become a prominent subject of investigation. An apparent inconsistency exists in many of its leading features, truth being mixed up with the rubbish of error. It conflicts with the prevailing opinions of the best informed medical men of the present age, and stands in direct opposition to the almost universal belief in the laws which govern epidemics, and determine the question of contagion, upon which false doctrine the laws of quarantine were originally based.

"My experience as regards our own quarantine system, has long since convinced me of the entire propriety of such a modification as shall place it upon an equality with the present improved state of the science of hygiene. The requirements of the law, as it now stands, are unnecessarily restrictive and burdensome, when enforced, as well as embarrassing to the commercial prosperity of our city, and should be remodelled.

"In advancing my own opinion on the question of a reform in quarantine, I am free to acknowledge, that while I desire a thorough change in the existing system, I am not an advocate for an entire repeal of those laws. My experience and observation lead me to this conclusion, however widely it may differ from the results arrived at by many of my learned countrymen, and the investigations and conclusions of enlightened commissions in Europe."

Mention is made of the opinions of influential men of earlier times, in favor of the abrogation of quarantine; as Maclean, Rush, Caldwell, Potter, and even President Jefferson, who, in 1804, "in a communication to Congress on the state of the Union, protested against the prevalence of a code of laws to prevent the introduction of yellow fever." But the weight of these and other authorities does not in the least shake the faith of the author of this address, as to the importance of a judicious code of quarantine restrictions. Not only commerce, but health must, of course, be duly considered in legislation.

"It is of paramount importance that the community be protected against the introduction of pestilential and contagious diseases, through the channels of commerce. This can only be done by an efficient system of quarantine laws; laws which, sufficiently restrictive in their operation, when properly enforced, to guard against the admission of disease, will not infringe unnecessarily upon the interests and rights of the owners, underwriters, or crews of vessels, when unfortunately detained, either by delays, expenses, or exposure and destruction of property; laws which will in nowise impede an active commerce, whose interests, thus jeoparded, have always been antagonistic to quarantine establishments."

The construction of a system which will, notwithstanding the presence of positive restrictions, reconcile fully the interests of commerce with those of public health, is indeed a great desideratum; it would seem to us, at present, a difficult one to hope for, under the name of *quarantine.*

It cannot be considered a fault, in the present address, that it does not discuss the details of the desired reformation in health laws; since such a topic was not proposed in its title, and would undoubtedly extend it too far. Dr. Jewell, in connection with the Philadelphia Board of Health, has, by recent correspondence throughout the country, obtained the calling of a Convention, of delegations "from the various boards of health in those cities interested, and from respectable medical organizations," to deliberate fully upon the whole subject; this conference "to receive the sanction of the State Legislatures, and in the end to have a national impress." The Convention, we are informed from another source, is to meet in this city on the 15th of May, 1857.

A very similar proposition to this was made by Dr. R. E. Griffith, thirty years ago, in the first number of the American Journal of Medical Sciences, Nov., 1827; looking toward the establishment, by delegates from the several States, of uniform health laws, adapted to the different localities throughout the Union.

We hope that this Convention may succeed in the object of its assemblage; but we must confess to some doubts of its perfect and immediate success, in settling forever the so vexed question of quarantine. We venture even to doubt whether its organization is upon the very best possible basis for present utility. It is to be composed of delegates from commercial bodies, from boards of health, *and* from medical societies; the medical element in its formation being evidently secondary. Can this afford *permanent* results? "While doctors disagree, who shall decide?" Until the scientific question is set at rest, how shall the practical one be disposed of? But, it may be inferred from the above programme, that, in the view of Dr. Jewell and others, medical men are not the most competent to decide upon the scientific data in question. This opinion, in fact, is not now uncommon. A writer in the British and Foreign Medico-Chirurgical Review, who has obtained a place in the literature of yellow fever (rather through the character of the journal than the merits of his article) in an *ex parte* review of Drs. La Roche and Barton, has broadly suggested what is here implied, namely, that medical men are not fit judges of medical evidence. This is done by him, it is true, in a very different spirit from that of Dr. Jewell; as he accuses of *partisanship* all those who differ from him; including, of course, the London General Board of Health, the Sanitary Commission of New Orleans, and all other bodies, and men, who, having concluded to disbelieve in the great efficacy of quarantine, *must*, therefore, be guilty of presenting "selected evidence." He would include, no doubt, in the same category, the illustrious Chervin himself, and the commission of eminent men appointed by the French Academy, to report upon his labors; who all deserved the epithet of partisan, certainly, as well as Dr. La Roche.

We may digress for a moment, to ask, what would such critics say in a similar relation, of Alexander von Humboldt? One might suppose that, in the whole history of science, there has never been a mind more fitted by its powers and culture, to test dispassionately just such a question as this. While in Mexico, Humboldt devoted himself to the subject of yellow fever, as one deserving of investigation; studied it as a naturalist; and what was the result? In his Political Essay, as is well known, he states, that "on the continent of equinoctial America, the *vomito* is no more contagious than the intermittent fevers of Europe."

In another profession, that of law,—whose special vocation it is to deal with evidence, and to sift certainty from semblance,—examples are not wanting of at least presumable impartiality in dealing with this subject. The reply of T. Jones Howell, Esq., Judge Advocate, to the Colonial Secretary of State, upon the epidemic at Gibraltar, in 1828,* affords an admirable instance; although it contains deductions adverse to quarantine.

But, we are not in doubt of the possibility of the Medical Profession itself arriving at certainty upon such points, *in time.* At least, it has sufficient power, rightly or wrongly, to unsettle any supposed certainty or

* Sec. Rep. on Quarantine, Eng. Board of Health, p. 245.

permanence otherwise attained. In France, and elsewhere, the question in regard to plague, cholera, and yellow fever, *has been* determined again and again; but, if not always disturbed afterwards, it has not afforded precedents for other quarters, and may hardly be considered, in any place, to have constituted a final settlement. Thus, in 1845, the Superior Council of Health, at Paris, presided over by the Minister of Commerce, unanimously pronounced that "science had arrived at a definite conclusion, and it was no longer allowable to believe in the contagious character of yellow fever!" Yet in the same place, seven years later, an International Sanitary Conference was held, which found it a part of its duty to *mitigate* quarantine restrictions, a very considerable stringency in which they still upheld.

It is unfortunate that differences in opinion amongst medical men, as amongst theologians, are constantly exaggerated, as well as maintained, by much greater differences in the use of phraseology. The remark of Pascal (quoted by Hosack), is quite apposite upon the subject of this notice. "*La différence qui est entre nous est si subtile, qu'à peine pouvons nous la marquer nous mêmes.*"

In the employment of the words *contagion* and *infection*, a world of confusion has existed, blending hypothesis with fact, and obscuring the real agreement of many controversialists, upon opposite sides, in regard to the essential matters of the question. That such an agreement has been, all along, underlying the warm dispute upon ambiguous terms, must be obvious to any one who will compare the language of those who have, by the energy of their argumentation, or the authority of their experience, attracted the most attention to their opinions upon yellow fever. It may not be unprofitable to exemplify this.

Thus, Currie, who is classed with the decided contagionists, admitted* that "a certain condition of the atmosphere must concur with the matter of contagion, before the disease can be communicated from one to another." Rush, notwithstanding his full recantation of the contagion theory, afterwards acknowledged, that matter capable of causing the disease might be "retained in beds or clothes," so as to give rise to cases of the fever even during the succeeding summer or autumn.†

Chisholm allows the local origination (from filth, &c.) of at least two epidemics, that of the Ship Hankey, off the African coast, and one in Demerara; although he distinguishes "the common fever of the West Indies" from the Bulam fever, in these and other particulars.‡

Hosack, who calls himself a believer in the contagion of yellow fever,

* Fever of 1799, p. 77.

† Rush's Inquiries, vol. iii, p. 103.

‡ Letter to Haygarth, p. 217; and Med. Repository, v. 229.

places it in his third class of communicable diseases,—to whose transmission "an impure atmosphere is *indispensably necessary*."*

Monette, an industrious contagionist, acknowledges the existence of sporadic cases, not communicating the disease, nor accounted for by such a mode of transmission.† Dr. Drake, who most laboriously investigated all the evidence in Western and Southwestern North America, and decided against contagion, yet states‡ that yellow fever cases have been produced "by exposure to the air escaped from goods sent from a city in which the disease was prevailing."

Dr. Dickson,§ although he considers yellow fever to be contagious, yet believes that, as a general rule, its contagiousness is *limited* by *certain contingencies;* that "it does not exist anywhere in winter," and that if carried into a cold region it will not diffuse itself.

Dr. E. H. Barton, one of the most able opponents of contagion, admits the occasional propagation of the disease by *fomites*,|| "in a kindred or congenial atmosphere." Dr. Barton also distinctly advocates quarantine; to which his fellow-citizen, Dr. Fenner, is less inclined, though an avowed "contingent contagionist."

Dr. W. P. Alison,¶ in one of the most philosophical articles which have appeared upon the side of the contagionists, asserts that such a mode of transmission can occur only in a *tainted district*, with *distinct boundaries*. He admits, also, the occasional origination of the fever on board of ships, without contagion or shore malaria; that this never takes place north of N. latitude, 48°, and that the contagion itself "cannot pass without destruction, over 1000 yards of water."

Lastly, Dr. McWilliam** considers that "the contagious properties which marked the Eclair fever at Boa Vista, were acquired," not essentially belonging to the disease.

If not from these opinions, thus displaying congruity in the midst of dissonance,—certainly from the facts on which they are based,—it might seem possible to draw a conclusion, and to make that conclusion so evidently the essential result of the facts upon which all agree, that no man could consistently give it his dissent. If the Convention, to which allusion has been made, can do this, it will do an immense service to the country and to the world. If it can even settle with clearness, correctness, and unanimity, the *terms* of the controversy, the logical conditions of the

* Copland's Med. Dictionary, Am. ed., vol. ii, p. 403.

† Drake's Principal Diseases, &c., 2d series, p. 270.

‡ Ibid. p. 298.

§ Elements of Medicine, p. 262.

|| Rep. of San. Commission, &c., p. 273–4; and p. 453.

¶ Brit. and For. Medico-Chirurg. Review, April, 1854.

** Report of the Fever at Boa Vista, p. 110.

problem, and the methods by which it is to be finally solved, it will yet do much; and this we believe to be all that ought to be expected of such an organization at the present moment.

We shall certainly be charged with didactic presumption, as well as with occupying too much valuable space, if we prolong this discussion by an attempt to give in a *coup d'œil*, what appears to us to be the present state of the question in regard to the communicability of yellow fever. Yet the temptation is great, and the occasion above-named may afford some excuse, even at the necessity of harping, for the thousandth time, upon familiar names and things.

First, then, as to terms. *What is contagion?* La Roche states that the profession has pretty much agreed upon a modification of the definition of Dr. Bayley; that it is "a poisonous material, capable of exciting a peculiar disease, and emanating, with that power, from a body sick with that disease." Dr. La Roche, himself, concentrates this elsewhere into the terms, "a specific, secreted poison." The word *secreted* may well be objected to; but its meaning here obviously is, *educed by a physiological or pathological process, such as can only occur in a living human body.*

Now, in this meaning, we venture to assert, that not a single fact has ever been made public, which warrants the conclusion that yellow fever has been proved to be contagious, under any circumstances whatever. There is but one "*instantia crucis*" of contagion; and that is, the transference of the disease, by inoculation, from person to person, under conditions forbidding any other origin for the second case. This has always failed with yellow fever. *The occasional personal communication of disease does not alone prove contagion.* We are obliged to differ, in holding this opinion, from Dr. La Roche.* The latter disbelieves, upon similar grounds, the contagiousness of typhus, of erysipelas, and of puerperal fever. His objections, however, to the supposition of occasional (however rare), transportation by persons, not through contagion, are chiefly *theoretical* objections, against the hypothesis associated with the term *fermentation;* an unnecessary hypothesis altogether. Notwithstanding his preference for the "vegetative decomposition" theory, Dr. La Roche admits the existence of a "peculiar poison," or "morbid material;" which, of course, must be *the* "conditio sine quà non," if it exist. Dr. L. disproves, also, the completeness of the "shears" theory of Dr. Barton,† by showing that, both the meteorological and the terrene conditions remaining the same, a locality may one year be affected with yellow fever, and another not. Dr. Barton acknowledges this, unconsciously, in admitting that the introduction of a case of the disease (all other circumstances remaining

* Vol. ii, p. 191. † Vol. ii, p. 109, and 623.

the same), into a congenial atmosphere, may propagate it.* Similar disproof of the "vegetative" or "ligneous" theory is abundant. The absence of the disease from the open country, generally, and from the Nile and the Ganges, in particular, suffice. Dr. J. Wilson has supplied what was wanting to this theory in the words, "*the peculiar influence known only by its effects, assumed to be essential in some way to the production of the disease.*" Call this a Poison, with Dr. Alison; how then is it propagated, how is it carried, how is it destroyed? These are the questions. Shall we venture to attempt to answer them? 1. It exists only at certain places. 2. Only at certain seasons. The meteorological and terrene conditions are well proved—if we include the latter within the wider generalization of vegetable decomposition, mostly with animal products also in abundance; but, these are *conditions* only, not sufficient causes, as above shown. The Poison, in question, displays a capriciousness or variability, in its law of increase and decrease, most suggestive of parallelism to organic life: especially when we remember that its main *habitat* is the tropics, and its period of growth and activity, that of the absence of frost. It is not necessary to pursue this hypothesis; no single objection can be urged against it, of force, except that it cannot yet be *proved.* This is the duty of time.

The question, then, seeming to be, not between the contagion and non-contagion of yellow fever, but as to how this morbid Poison is transmitted, we may state that the general rule is proved to be,—its extension in the manner of an epidemic or endemic only. The range of this may be sometimes very limited; as in the instance of sporadic cases—abundantly upon record. We could, if desirable, burden these pages with numerous references upon this point; but it has been already done. The latest evidence of interest in regard to it, is to be found in the recent pamphlet of Dr. Arnold, of Savannah, based upon accurate and extensive experience in the post-mortem appearances of the disease.†

But, certain very credible authorities positively state facts which are difficult to explain, unless upon the theory of occasional personal transmission. It does not seem to us fair to deny all of these facts. It may be remarked upon them, however, 1. That some of them have been already, with great probability, otherwise explained. This is very curiously shown, for example, in the report of Dr. N. B. Benedict, to the Sanitary Commission,‡ in regard to an epidemic at Hollywood, in Alabama, in 1853.

2. That such cases are, to make the very best of them, *exceedingly rare.*

3. That the extension of the disease from them has never been wide;

* Rep. of Sanitary Commission, &c., p. 264.

† Essay upon the Relation of Bilious and Yellow Fever; Addendum, p. 6.

‡ Report of Sanitary Commission of New Orleans, p. 123.

scarcely ever reaching to more than one or two individuals. The recognized theory of *ship infection* (vessels affording the local causes), will account for supposed examples to the contrary. What, then, it will be asked, do we say of the famous "modern instances?" Of Ascension, Boa Vista, Fernando Po, and Goree? Only, that the best of them, so considered, that of Boa Vista, is vitiated, radically, by the length of *time* in the dates—the broken link at the case of Anna Gallinha, and the fact that yellow fever was already existing at the neighboring island of St. Jago, without any contagion. Even in the numerous instances of infection from ships, the disorder has rarely extended many rods. Of this, perhaps, no better or more interesting example can be afforded than that of Fort Hamilton, near Brooklyn, N. Y., in 1856.

We may ask, then, next, how is this Poison *destroyed?* 1. Always by freezing weather. 2. In *almost* every case, by the removal or absence of *filth*, and of *newly upturned soil.* The qualification named is necessary, in accordance with the present state of facts and assertions. It may be done away with, probably, hereafter. 3. In the language of Alison (contagionist), "North of 48° lat., at least, it cannot pass over a thousand yards of water without being deprived of its power."* In the words of Dr. C. H. Stone, of Natchez,† "*it cannot remain against a ten knot breeze.*"

4. There is no adequate reason, whatever, for believing that it attaches, especially to, or is multiplied in, the bodies of the sick.

It cannot be supposed possible, in case it should be connected with the person of an individual, or with clothing, bedding, or merchandise, that it would fail to be dissipated or rendered inert by cleansing and disinfecting processes of the ordinary kind; and the same may be asserted, unhesitatingly, in regard to ships.

5. Removal of the inhabitants from the locality of the infection will, in all instances, cause it to die out.

But we are transgressing all reasonable limits. Cutting short our exploration of a boundless field, we must allude only to the fact that Baltimore, in 1819, modified her quarantine laws, so as to allow *passengers*, in all cases, to go freely; and yet, she has had no epidemic of yellow fever since; notwithstanding close intercourse with infected ports, and especially with those of Norfolk and Portsmouth, in 1855.

In conclusion, in place of an expression of individual opinion upon the subject originally entered on in this review, we prefer to quote the language used by Dr. Griffith, thirty years ago, in the paper already mentioned.‡

"All vessels from tropical climates, whether from healthy or unhealthy ports, should be obliged to stop at quarantine until they have been cleansed and venti-

---

* Brit. and For. Medico-Chirurg. Rev., April, 1854.

† Trans. Am. Med. Assoc., 1856. ‡ Am. Jour. of Med. Sci., Nov. 1827.

lated, and the state of their cargoes examined; if these are found in an unsound and putrefying state, they should be discharged, and the unsound parts separated and detained, whilst the sound, after having been purified, might be permitted to proceed to the city. After a vessel has been discharged in port, she should undergo a thorough and complete ventilation and purifying, under the inspection of an officer appointed for that purpose.

"Passengers should be permitted to leave quarantine, unless when laboring under disease.

"The boards should be endowed with full powers, on the appearance of a contagious or malignant disease, to remove all those attacked, and if necessary, to vacate and prohibit all intercourse with that part of the town.

"They should have the power to cause to be removed all nuisances, which might tend to the deterioration of the health of the city . . . . . . . . the wharves should also be paved and regularly cleansed, and the docks should be of such a depth as never to be uncovered during low tides."

